# Scratching the Surface of Individual Aerosol Particle Properties

**DOI:** 10.1021/acscentsci.3c01362

**Published:** 2023-11-14

**Authors:** Andrew P. Ault, Cara M. Waters

**Affiliations:** Department of Chemistry, University of Michigan, Ann Arbor, Michigan 48109, United States

Atmospheric aerosols have wide-ranging impacts on global climate through their ability to nucleate cloud droplets and ice crystals as well as scatter and absorb solar radiation. Together these direct and indirect effects are the reason that aerosols represent the greatest uncertainty within earth’s radiative budget (i.e., global warming).^1^ To illustrate the importance of aerosols on climate, consider the fact that every cloud droplet in earth’s atmosphere nucleates on an existing aerosol particle, as our atmosphere never reaches relative humidities high enough to nucleate pure water vapor.^2^ However, not all particles are created equal, and like a snowflake, each individual particle in the atmospheric has a unique chemical composition and set of physical properties, which determine its ability to take up water, form cloud droplets, and ultimately impact climate. This is particularly challenging as the most common size for an atmospheric particle is ∼100 nm, and the most important sizes for aerosol-climate effects through nucleating cloud droplets and ice crystals or scattering and absorbing solar radiation are tens of nanometers to a few micrometers in diameter. Traditional aerosol measurements have gotten around the limited mass of these nano-to-micrometer-sized particles, with masses of femtograms to picograms per particle, by analyzing millions to billions or more particles together (e.g., extracts from filters) and assuming that particle chemical composition and physical properties can be treated as an ensemble average analogous to bulk properties in a beaker.^3^ Measuring an ensemble average to determine the behavior of individual particles loses key details of particle-to-particle variability, which would be analogous to melting a snowball and then trying to figure out the shape of the original snowflakes afterward. Understanding the chemical and physical properties of a population of individual particles, often referred to as the aerosol mixing state, is essential to link to the global-scale impacts of aerosols on clouds and climate. Although measuring the chemical composition of a nano-to-micrometer-sized particle is feasible,^3^ measuring other properties such as the surface tension for individual suspended particles in that same size range has remained a challenge. In this issue of *ACS Central Science*, Bzdek, Prisle, and co-workers highlight the difference in surface tension of levitated particles (via optical tweezing) as a function of size and surfactant.^4^ They also highlight the challenges traditional surface-tension models have in accounting for these differences and work with modelers to improve model representations. A key finding from their work is that micrometer-sized particles have higher surface tensions as particle size decreases.

**Figure 1:**
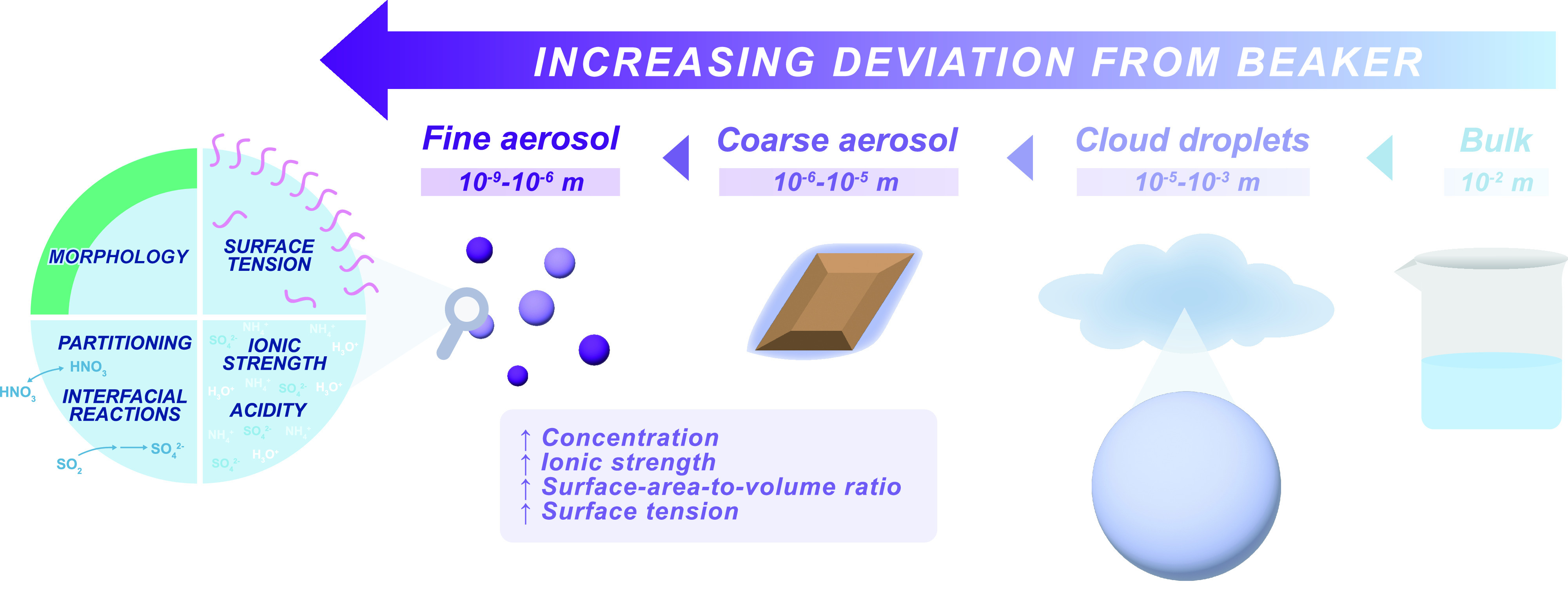
Diagram highlighting the sizes of fine and coarse aerosols versus cloud droplets and beakers (right). Physical and chemical differences of fine aerosol (left) are highlighted, which often differ from beaker-scale observations of bulk solutions.

To appreciate the impacts of aerosol size on chemical properties, such as the surface tension effects in Bain et al.,^4^ it is helpful to consider how small atmospheric aerosols are in comparison to the beakers in which we make many bulk chemical measurements. Figure 1 shows the size of a beaker, a cloud droplet, a coarse mode particle, and a fine mode particle. A fine mode particle has a diameter that is 10^4^–10^7^ times smaller than a solution in a beaker as well as no barrier (e.g., glass) to inhibit interaction with the surrounding atmosphere. This leads to some key differences between aerosols and solutions in beakers, which include increased surface-to-volume ratio, increased concentration/ionic strength due to lower water content, and elevated surface tension.

For a nano-to-micrometer-sized aerosol particle, it is not uncommon to have ionic strengths >10 M, which leads to extreme activity coefficients and shifted acid–base equilibria compared to dilute solutions. Furthermore, fine aerosol particles frequently do not exist at thermodynamic equilibrium in the atmosphere but rather exist in metastable states where the particle would thermodynamically prefer to effloresce to a solid (i.e., crystallize), but it exists as a supersaturated solution until it can overcome the energy barrier to forming a new phase.^5^ This can lead to hysteresis effects of greater than 40% relative humidity between deliquescence (solid to liquid transition) and efflorescence (liquid to solid transition). In the high-salt environment of an atmospheric aerosol particle, organic species are often no longer miscible and salt out to form a liquid–liquid phase-separated particle with an organic shell and an aqueous core.^6^ The high-salt environment can also modify gas-particle partitioning, causing further deviation from an ideal, dilute system.^7^ The pH of atmospheric aerosols is also dynamic and challenging to measure,^8^ though emerging single-particle and bulk measurements have shown more acidic aerosol at smaller sizes for aerosol generated from the same solution.^8^ Finally, within the atmosphere, particle–gas interfaces can also represent complicated environments in the first few monolayers of a particle, which can lead to accelerated reaction kinetics on aqueous particles in comparison to the same reaction in a beaker.^9^ Thus, in the extreme environment of an atmospheric aerosol particle, the behavior of surface tension, ionic strength, acidity, acid–base equilibria, partitioning, and interfacial reactions frequently deviates from our chemical intuition based on a beaker-scale understanding.

To fully appreciate the importance of surface tension specifically, as probed in Bain et al.,^4^ it is worth considering the impacts of aerosols on clouds and climate mentioned above. The ability of an individual particle to take up water and form a cloud droplet is often represented using Kappa-Köhler theory,^10^ with the key equation being
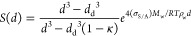
where *S* is the supersaturation ratio, *d* is the diameter of the droplet, *d*_d_ is the dry diameter of the particle, κ is the hygroscopicity parameter, *M*_w_ is the molecular weight of water, *R* is the gas constant, *T* is temperature, ρ_w_ is the density of water, and, most importantly, σ_S/A_ is the surface tension at the air–water interface. Whereas extensive aerosol chemistry research has focused on understanding hygroscopicity via the κ term, comparatively little research has focused on surface tension at the air–water interface. This has been due in part to the difficulty in measuring surface tension for suspended particles at atmospherically relevant sizes. Simply using a tensiometer or other macroscale surface tension measurement will not provide the needed understanding of aerosol surface tension for micrometer-sized and smaller particles. Thus, the work in Bain et al.^4^ is a significant step forward for improving the chemical understanding of surface tension at the air–water interface with broad implications for chemistry and climate. It ties into the broader need to understand the properties of individual particles and the impacts that these have on aerosol chemistry and, ultimately, aerosol impacts on climate.
